# Association of Spinal Corrective Surgery With Abdominal Aorta Length in Patients With Adult Spinal Deformity

**DOI:** 10.7759/cureus.56341

**Published:** 2024-03-17

**Authors:** Shuhei Ohyama, Toshiaki Kotani, Yasushi Iijima, Takahiro Sunami, Shun Okuwaki, Tsuyoshi Sakuma, Yosuke Ogata, Shuhei Iwata, Tsutomu Akazawa, Kazuhide Inage, Yasuhiro Shiga, Shohei Minami, Seiji Ohtori

**Affiliations:** 1 Department of Orthopedic Surgery, Seirei Sakura Citizen Hospital, Sakura, JPN; 2 Department of Orthopedic Surgery, University of Tsukuba, Tsukuba, JPN; 3 Department of Orthopedic Surgery, St. Marianna University School of Medicine, Kawasaki, JPN; 4 Department of Orthopedic Surgery, Chiba University, Graduate School of Medicine, Chiba, JPN

**Keywords:** spinal sagittal alignment, abdominal aorta length, aortic elongation, adult spinal deformity, spinal corrective surgery

## Abstract

Introduction

This research aimed to explore the relationship between spinal characteristics and the length of the abdominal aorta in adult spinal deformity (ASD) patients who underwent corrective spinal surgery. We hypothesized that adjusting spinal alignment might affect the abdominal aorta's length.

Methods

This study included thirteen patients with ASD (average age: 63.0 ± 8.9 years; four males and nine females) who received spinal correction surgery. We measured both pre-operative and post-operative spinal parameters, including thoracolumbar kyphosis (TLK), and calculated their differences (Δ). The length of the aorta (AoL) was determined using an automated process that measures the central luminal line from the celiac artery's bifurcation to the inferior mesenteric artery. This measurement was made using contrast-enhanced computed tomography for three-dimensional aortic reconstruction. We compared the pre-operative and post-operative AoLs and their differences (Δ). The study examined the correlation between changes in spinal parameters and changes in AoL.

Results

Post-operatively, there was an increase in aortic length (ΔAoL: 4.2 ± 4.9 mm). There was a negative correlation between the change in TLK and the change in AoL (R^2 ^= 0.45, p = 0.012, β = −0.21). No significant correlations were found with other spinal parameters.

Conclusions

The abdominal aorta can elongate by 4.8% after spinal corrective surgery in patients with ASD. The degree of elongation of the abdominal aorta is associated with spinal alignment correction.

## Introduction

Previous studies have reported the favorable outcomes of spinal corrective surgery in patients with adult spinal deformity (ASD) [1,2]. A combination of several techniques can be used to achieve appropriate spinal alignment, including lateral interbody fusion (LIF) and spinal osteotomy, the latter being particularly useful in patients with rigid deformities [3-5].

Despite the positive post-operative results, these procedures can cause devastating complications due to iatrogenic aortic injury during spinal surgery, caused by surgical instruments or by significantly affecting aortic elongation [6-8]. Previous reports of aortic injury in patients who underwent extensive spinal alignment support the idea that high aortic elongation forces may be an important cause of vascular damage [7,8]. Thus, several studies have analyzed the positional relationship between the spine and the aorta and how this positional relationship changes after spinal corrective surgery [6,9,10]. However, few studies have considered how changes in spinal alignment affect aorta elongation [11,12]. Most of these studies have measured the aortic length (AoL) after spinal corrective surgery using a linear approximation based on the vertebral bodies [11,12]. The main disadvantage of this method is that the AoL may not be measured accurately, especially in the setting of complex deformations of the aortic location or spine. In the field of cardiovascular surgery, measurement of aortic length and the cross-sectional area of the aorta using three-dimensional aortic reconstruction with contrast-enhanced computed tomography (CT) has been shown to provide accurate results [13,14].

In this study, we measured AoL using three-dimensional aortic reconstruction with contrast-enhanced CT in patients undergoing spinal corrective surgery. We aimed to determine the degree of aortic elongation after surgery and to study the association of spinal alignment with AoL.

## Materials and methods

Study population

From June 2016 to September 2022, data from consecutive patients with ASD who underwent combined anterior-posterior spinal fusion surgery in our institution was gathered. Adult spinal deformity was defined as the presence of at least one of the following indicators: degenerative or idiopathic scoliosis with spinal curvature >20° in the coronal plane, C7 sagittal vertical axis (SVA) >50 mm, pelvic tilt (PT) >25°, and T5-T12 thoracic kyphosis (TK) > 60° [15]. Patients with available preoperative and postoperative contrast-enhanced CT were included, whereas patients whose CT couldn’t evaluate the abdominal aorta were excluded.

The study adhered to the guidelines of the Declaration of Helsinki, and the study protocol was approved by the institutional review board. Written informed consent was obtained from all participants.

Patient data

We reviewed patients' demographic information, such as age, sex, and body mass index (BMI). Spinal parameters, including coronal Cobb angle, SVA, TK, thoracolumbar kyphosis (TLK; T10-L2), lumbar lordosis (LL), PT, and pelvic incidence (PI), were measured using preoperative and postoperative whole-spine lateral radiographs. Changes (Δ) in spinal parameters were determined by subtracting preoperative values from postoperative values. To evaluate the calcification of the abdominal aorta, preoperative abdominal aortic calcification (AAC) scores of the anterior and posterior walls of the abdominal aorta over L1 to L4 were recorded. The calcification score of each section ranges from 0 to 3. Score zero means with no calcification; score one means calcification length less than 1/3 of the vertebral body; score two means the calcification length spanned from 1/3 to 2/3 of the vertebral body; score three means calcification length greater than 2/3 of the vertebral body. AAC score is the sum of calcification scores from L1 to L4 with a maximum of 24 [16].

**Figure 1:**
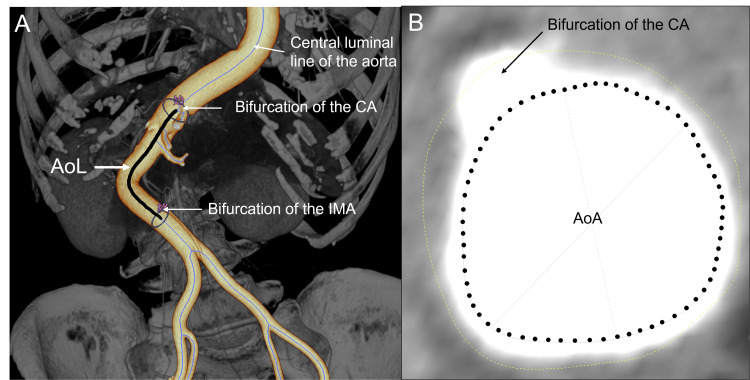
Measurement of the aortic length (AoL) and cross-sectional area of the aorta (AoA) (A) Determination of the aortic length (AoL; black line). (B) Determination of the cross-sectional area of the aorta (AoA; dotted circle). CA: celiac artery; IMA: inferior mesenteric artery.

Preoperative and postoperative abdominal aorta imaging was conducted using 64-slice multidetector computed tomography (MDCT) (Revolution HD, GE Healthcare Japan). For preoperative assessment, CT scans were taken in the lateral decubitus position to facilitate LIF technique preparation, ensuring accurate identification of major vessels and the ureter. Postoperative scans were performed with patients in a supine position. The CT scan parameters included a tube voltage of 120 kVp, a tube current ranging from 200-575 mAs, a pitch of 0.984, a pantry rotation time of 0.8 seconds, a slice thickness of 1.25 mm, a reconstruction interval of 0.625, a detector collimation of 64 × 0.625, and a field of view of 32 cm. A 70-100 mL volume of non-ionic contrast agent was administered at a rate of 3-4 mL/sec using a pressure injector, followed by a saline flush. All MDCT source images were transferred to our hospital’s picture archiving and communication system (PACS). Early arterial phase CT images with a 1.25  mm slice thickness were transferred to a 3D image analysis workstation (SYNAPSE VINCENT, Version 4.1, Fujifilm Co., Tokyo, Japan) for evaluation. The central luminal line of the aorta was automatically traced. The AoL was defined as the length of this line from the bifurcation of the celiac artery (CA) to the inferior mesenteric artery (IMA) (Figure 1A). This length was automatically calculated (Video 1) [13,14]. The AoA was determined as the cross-sectional area at the CA bifurcation, also calculated automatically (Figure 1B). Changes in AoL and AoA (ΔAoL and ΔAoA) were calculated based on the differences between preoperative and postoperative values. 

**Video 1:** Measurement of aortic length (AoL). Aortic length (AoL) was determined as the length of the center luminal line of the aorta between the bifurcation both of the celiac artery (CA) and the inferior mesenteric artery (IMA).

To investigate the influence of spinal parameters on ΔAoL, a univariate regression analysis was performed between spinal parameters and ΔAoL. When a P-value (p) < 0.2 was observed in a univariate regression analysis, clinically significant variables were included in a multiple regression analysis. To manage multicollinearity, variants with a variance inflation factor >5 were removed. To examine the influence of ΔAoL on ΔAoA, a univariate regression analysis was performed between ΔAoL and ΔAoA.

AoL and AoA values could be different for each observer because the slice settings for the bifurcation of the CA and IMA were done manually. To evaluate intra- and inter-observer reliability, AoL was assessed from CT images by two spinal surgeons. The observer reviewed the same patient series in a second session. Reproducibility was evaluated using intraclass correlation coefficients (ICC).

Statistical analysis

Values were expressed as the mean ± standard deviation (SD). Linear regression analysis was used to investigate the correlations between spinal parameters and ΔAoL and between ΔAoL and ΔAoA. Variables were included in a multiple regression analysis when a p < 0.2 was observed from the simple regression analysis. Statistical analysis was performed using SPSS version 25 (IBM, Armonk, NY, USA), with the significance level set at p < 0.05.

## Results

Patient data

Data from 15 patients were collected, but data from two patients whose CT could not evaluate the abdominal aorta were excluded. In total, 13 patients (63.0 ± 8.9 years (range: 43-76 years); four males/nine females; BMI 22.1 ± 2.4 kg/m^2^) were included in this study. One patient was treated by pedicle subtraction osteotomy (PSO), and the others were treated with the LIF technique without spinal osteotomy. The intra- and inter-observer reliabilities of measurement of AoL and AoA were within acceptable limits (ICC = 0.97 and 0.88, respectively). Comparison of parameters pre- and post-spinal surgery showed significant differences (Table 1). The ACC score was 2.5 ± 6.0. One patient had a score of 24, and all others had a score of less than five. Another eight patients had a score of zero.

**Figure 2:**
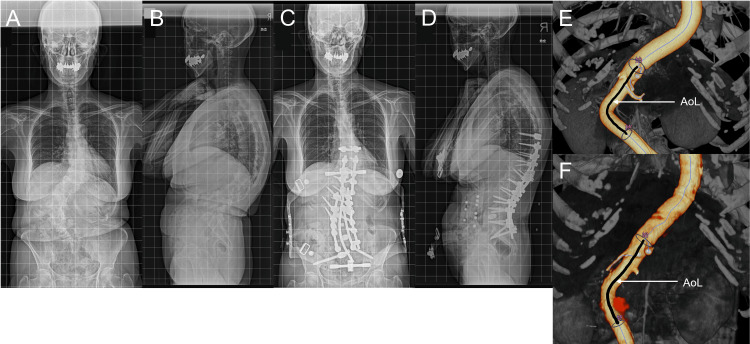
Illustrative case of a 67-year-old female with adult spinal deformity treated with posterior corrective fusion and multilevel lateral interbody fusion technique. (A), (B), and (E): Pre-operative images. (C), (D), and (F): Post-operative images.

**Figure 3:**
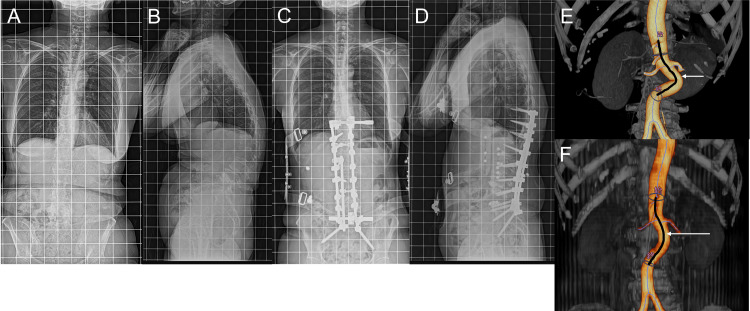
Illustrative case of a 57-year-old female with adult spinal deformity treated with posterior corrective fusion, pedicle subtraction osteotomy, and multilevel lateral interbody fusion technique. (A), (B), and (E): Pre-operative images. (C), (D), and (F): Post-operative images.

As shown in Table 1, the ΔAoL was 4.2 ± 4.9 mm, suggesting that the abdominal aorta between the bifurcation of both the CA and IMA elongated by 4.8% after spinal corrective surgery. On the contrary, AoA decreased by 6.9% after spinal corrective surgery. Moreover, Figure 2 and Figure 3 exemplify these changes in patients who underwent multilevel LIF and PSO with multilevel LIF, respectively. 

**Table 1:** Pre-operative and post-operative spinal parameters, AoL, and AoA. Values are presented as the mean ± standard deviation. Δ: difference obtained by subtracting pre-operative from post-operative values, SVA: sagittal vertical axis, TK: thoracic kyphosis, TLK: thoracolumbar kyphosis, LL: lumbar lordosis (L1-S1), PT: pelvic tilt, PI: pelvic incidence, AoL: aortic length between the bifurcation of the celiac artery and the bifurcation of the inferior mesenteric artery, AoA: cross-sectional area of the aorta at the bifurcation of the celiac artery.

	Pre-operative	Post-operative	Δ
Coronal Cobb (°)	43.9 ± 22.3	19.7 ± 11.6	–24.2 ± 13.9
SVA (°)	6.7 ± 5.3	1.7 ± 2.4	–5.0 ± 5.1
TK (°)	22.5 ± 14.3	30.0 ± 10.2	7.5 ± 10.2
TLK (°)	28.3 ± 14.0	10.3 ± 7.5	–18.0 ± 15.5
LL (°)	20.6 ± 15.0	44.6 ± 10.8	24.0 ± 17.3
PT (°)	30.8 ± 11.3	18.4 ± 6.8	–12.5 ± 9.9
PI (°)	48.7 ± 15.8	48.9 ± 13.3	0.2 ± 6.9
PI-LL (°)	28.1 ± 19.2	4.3 ± 9.9	–23.8 ± 16.5
AoL (mm)	88.4 ± 9.4	92.6 ± 8.7	4.2 ± 4.9
AoA (mm^2^)	272.5 ± 47.5	253.6 ± 45.8	–18.9 ± 44.2

The variables showing p < 0.2 in the univariate regression analysis were Δcoronal Cobb and ΔTLK. Therefore, the multivariate model was constructed with Δcoronal Cobb and ΔTLK (Table 2). The multivariate regression analysis showed that ΔTLK negatively correlated with ΔAoL (R^2^ = 0.45, adjusted R^2^ = 0.40, p = 0.012, β = −0.21). There was no significant correlation between other spinal parameters and ΔAoL (Table 2). There was also no significant correlation between the ACC score and ΔAoL.

**Table 2:** Correlation between Δspinal parameters and ΔAoL. *Indicates statistically significant differences. Δ: difference obtained by subtracting pre-operative from post-operative values; AoL: aortic length between the bifurcation of the celiac artery and bifurcation of the inferior mesenteric artery; CI: confidence interval; SVA: sagittal vertical axis, TK: thoracic kyphosis, TLK: thoracolumbar kyphosis, LL: lumbar lordosis (L1-S1), PT: pelvic tilt, PI: pelvic incidence.

Dependent variable	Independent variables	Standardized regression coefficient	95% CI		p-value
			Lower	Upper	
ΔAoL	Δcoronal Cobb	0.15	–0.068	0.36	0.16
	ΔSVA	0.20	-0.42	0.83	0.49
	ΔTK	–0.14	–0.45	0.16	0.33
	ΔTLK	–0.21	–0.37	–0.058	0.012*
	ΔLL	0.049	–0.14	0.24	0.57
	ΔPT	–0.064	–0.39	0.26	0.68
	ΔPI-LL	–0.055	–0.25	0.14	0.55

ΔAoL correlated negatively with ΔAoA (R^2^ = 0.54, adjusted R^2^ = 0.50, p = 0.004, β = 0.74), suggesting that the more the aorta was elongated, the smaller the cross-sectional area of the aorta became. The intra- and inter-observer reliabilities of measurement of AoL and AoA were within acceptable limits (ICC = 0.97 and 0.88, respectively).

## Discussion

Our study showed that the abdominal aorta elongated by 4.8% after spinal corrective surgery in patients with ASD. In this study, AoA decreased after surgery and ΔAoA negatively correlated with ΔAoL. These results suggest an elastic strain of the aorta after surgery; the aorta extended vertically and became thinner after surgery. In other words, spinal corrective surgery subjects the aorta to an elongation force, changing its length. Our results are consistent with previous studies and provide further evidence of this association with a more accurate methodology.

**Figure 4:**
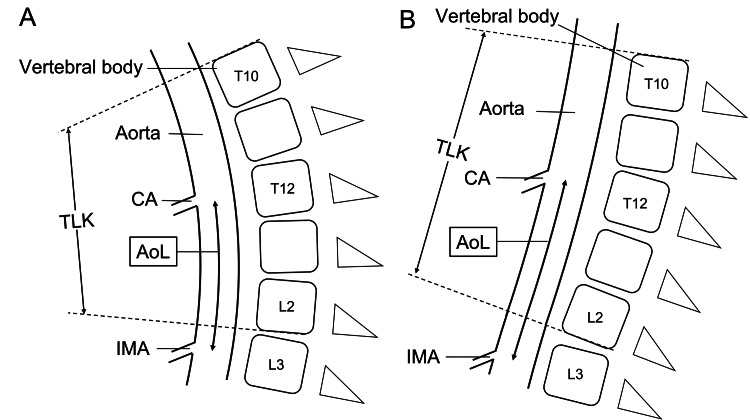
Schematic illustrations of the mechanism of aortic length (AoL) changes in the context of thoracolumbar kyphosis (TLK). (A): Pre-operative. (B): Post-operative. CA: celiac artery, IMA: inferior mesenteric artery.

Interestingly, the length of the aorta between the bifurcation of both the CA and IMA correlated with the correction of TLK. The CA usually arises at the height of the T12 vertebral body, and the IMA usually arises at the height of the L3 vertebral body [17-19]. Thus, the correction of the thoracolumbar spine between the T12 and L3 vertebral bodies can influence the morphology of the abdominal aorta between the bifurcation of the CA and the bifurcation of the IMA (Figure 4). Based on the results of this study, it is conceivable that spinal alignments other than TLK, such as LL and TK, could also be related to aortic length if the range of aortic length measurement were wider. However, no clear correlation was found in this study. Further studies will be needed.

**Figure 5:**
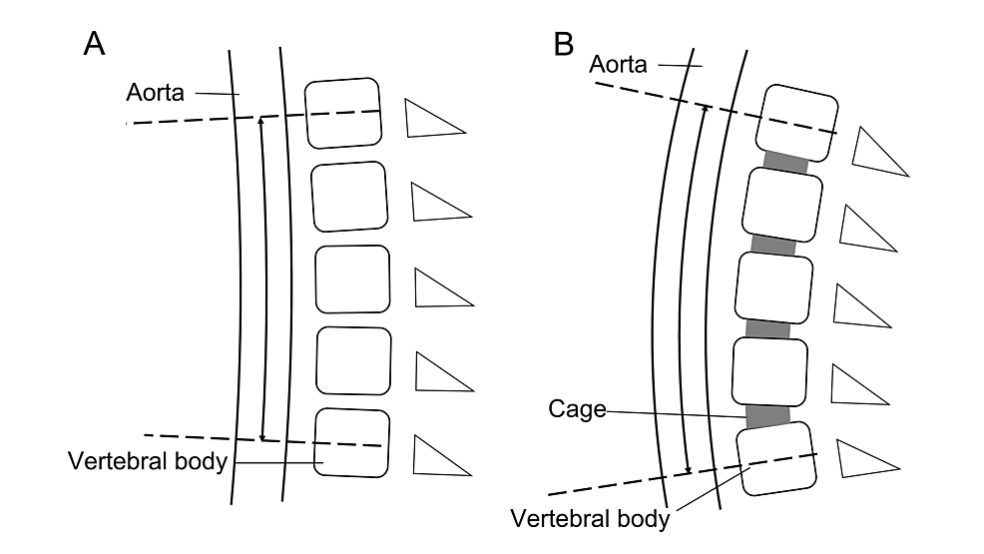
Schematic illustrations of the change in length of a portion of the aorta (double arrow) in a case treated with the multilevel lateral interbody fusion technique. (A): Pre-operative. (B): Post-operative.

**Figure 6:**
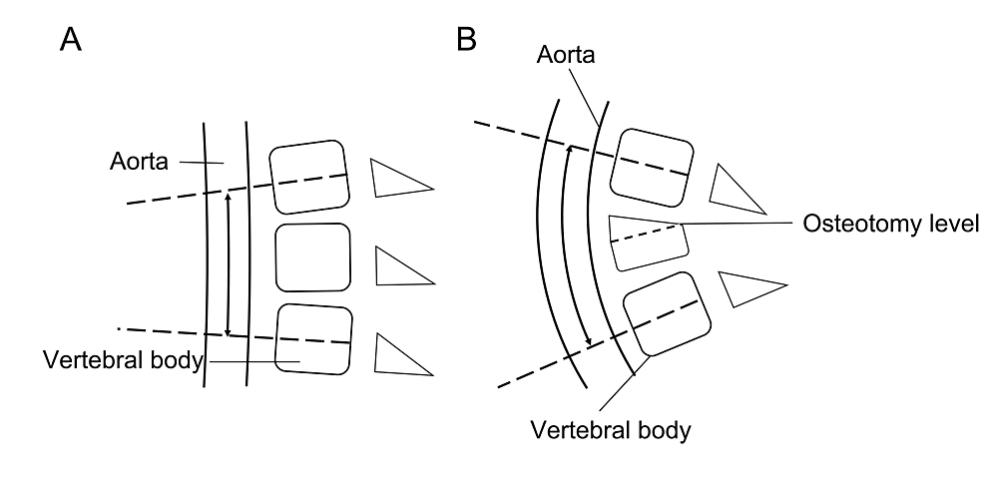
Schematic illustrations of the change in length of a portion of the aorta (double arrow) in a case treated with spinal osteotomy. (A): Pre-operative. (B): Post-operative.

Aortic rupture can occur when the aortic wall is elongated to 140% of its original length [20]. In this study, the abdominal aorta was elongated by 4.8% after surgery. The possibility of aortic rupture due to aortic elongation associated with multilevel lateral interbody fusion surgery is rather small (Figure 5). However, calcification of the abdominal aorta affects the condition of the aortic wall [21]. In this study, calcification was not related to the degree of aortic elongation, but calcification and increasing age are closely related to the deterioration of the stiffness and strength of the aortic wall [21]. Even if aortic elongation is relatively small, there is a risk of aortic rupture in spinal corrective surgery for patients with aortic calcification. On the other hand, extremely large local corrections, including PSO and vertebral column resection, may be associated with extreme aortic elongation in a narrow segment of the aorta (Figure 6). However, there was only one case of PSO in this study, and further studies are needed to determine whether a large local correction is associated with greater aortic elongation.

This study has several limitations. First, the number of patients enrolled was small. Second, the range of the length of the aorta that we measured was limited; therefore, we can only draw conclusions regarding the relationship between the length of the aorta and spinal parameters locally. However, contrast-enhanced CT was performed only for patients with post-operative abdominal symptoms and was not aimed to evaluate the whole aorta. Third, this study did not evaluate the degree of spinal correction that can cause aortic injury. Fourth, the body position of the CT scan differs between pre-operative and post-operative. The difference in body position may affect the anatomic positioning of the spine and abdominal aorta. Fifth, this study did not compare the impact of spinal osteotomy with the LIF technique in relation to changes in the length of the aorta. 

## Conclusions

The abdominal aorta can elongate by 4.8% after spinal corrective surgery in patients with ASD. The degree of elongation of the abdominal aorta was associated with the correction of spinal alignment. Extremely large local corrections of spinal kyphosis might cause aortic injury during spinal corrective surgery.
